# Repurposing of an immunosuppressive drug holds promise for stem-cell-based neurorepair

**Published:** 2014-08

Injury and diseases of the central nervous system can have a devastating effect on quality of life. Given recent progress in regenerative medicine, there is increasing interest in the development of therapies that regenerate or repair damaged nerve tissue to enable functional recovery. Strategies that aim to harness the regenerative potential of resident neural precursor cells (NPCs) are particularly promising. Recently, it has been shown that an FDA-approved immunosuppressive drug, cyclosporin A (CsA), can potentiate NPC survival. Interestingly, it has direct effects on NPCs and the pro-survival effects are not mediated by immunosuppression. Here, Cindi Morshead and co-authors use an elegant *in vitro* system based on cultured neurospheres to elucidate the molecular mechanism by which CsA promotes NPC survival. CsA’s immunosuppressive effects are mediated by a calcium-regulated phosphatase, calcineurin. Treatment of neurospheres with an inhibitor of calcineurin had no effect on NPC survival, whereas a non-immunosuppressive analogue of CsA (NIM811) is able to mimic the pro-survival effect of the drug. NIM811 can also promote functional recovery in a mouse model of stroke. These data provide molecular evidence that CsA enhances NPC survival without triggering immunosuppressive pathways, supporting the repurposing of this drug in novel regenerative therapies for CNS disease or injury. **Page 953**

